# Association Between Young-Onset Dementia and Risk of Hospitalization for Motor Vehicle Crash Injury in Taiwan

**DOI:** 10.1001/jamanetworkopen.2022.10474

**Published:** 2022-05-05

**Authors:** Chih-Ching Liu, Chien-Hui Liu, Kun-Chia Chang, Ming-Chung Ko, Pei-Chen Lee, Jiun-Yi Wang

**Affiliations:** 1 Department of Healthcare Administration, College of Medical and Health Science, Asia University, Taichung, Taiwan; 2 School of Nursing, National Yang Ming Chiao Tung University, Hsinchu, Taiwan; 3 Jianan Psychiatric Center, Ministry of Health and Welfare, Tainan, Taiwan; 4 Department of Natural Biotechnology, NanHua University, Chiayi, Taiwan; 5 Department of Surgery, Zhong-Xing Branch, Taipei City Hospital, Taipei, Taiwan; 6 Department of Health Care Management, National Taipei University of Nursing and Health Sciences, Taipei, Taiwan; 7 Department of Medical Research, China Medical University Hospital, China Medical University, Taichung, Taiwan

## Abstract

**Importance:**

Several studies have suggested that older-onset dementia is associated with an increased risk of motor vehicle crash injury (MVCI). However, evidence of an association between young-onset dementia and the risk of MVCI is insufficient, particularly in Asia.

**Objective:**

To investigate the association between young-onset dementia and MVCI-related hospitalization in Taiwan.

**Design, Setting, and Participants:**

In this nationwide, population-based cohort study in Taiwan, a cohort of 39 344 patients aged 40 to 64 years with incident dementia diagnosed between 2006 and 2012 was matched 1:1 with a cohort of participants without dementia by age, sex, and index year (initial diagnosis of dementia). Participants were identified from Taiwan’s National Health Insurance Research Database (NHIRD). Data were analyzed between March 25 and October 22, 2021.

**Exposures:**

Dementia, defined by *International Classification of Diseases, Ninth Revision, Clinical Modification* codes.

**Main Outcomes and Measures:**

Hospitalization for MVCI, determined using linked data from Taiwan’s Police-Reported Traffic Accident Registry and the NHIRD from January 1, 2003, to December 31, 2015. Hazard ratios (HRs) for MVCI-related hospitalization were estimated using Cox proportional hazards regression models adjusted for sex, age, salary-based insurance premium, urbanization level, and comorbidities.

**Results:**

Of the 78 688 participants, 47 034 (59.8%) were male; the mean (SD) age was 54.5 (7.4) years. During the 10-year follow-up period, the incidence density of MVCI-related hospitalization was 45.58 per 10 000 person-years (95% CI, 42.77-48.39 per 10 000 person-years) among participants with dementia and 24.10 per 10 000 person-years (95% CI, 22.22-25.99 per 10 000 person-years) among participants without dementia. Compared with participants without dementia, patients with young-onset dementia were at higher risk of MVCI-related hospitalization (adjusted HR [aHR], 1.83; 95% CI, 1.63-2.06), especially those in younger age groups (aged 40-44 years: aHR, 3.54; 95% CI, 2.48-5.07) and within a shorter period (within 1 year of follow-up: aHR, 3.53; 95% CI, 2.50-4.98) after dementia was diagnosed. Patients with young-onset dementia also had a higher risk of being a pedestrian when the crash occurred (aHR, 2.89; 95% CI, 2.04-4.11), having an intracranial or internal injury (aHR, 2.44; 95% CI, 2.02-2.94), and having a severe injury (aHR, 2.90; 95% CI, 2.16-3.89).

**Conclusions and Relevance:**

In this retrospective cohort study, patients in Taiwan with a diagnosis of young-onset dementia had a higher risk of MVCI-related hospitalization than did individuals without dementia and the risk varied by age, disease duration, transport mode, injury type, and injury severity. These findings suggest a need for the planning of strategies to prevent transportation crashes among patients with young-onset dementia.

## Key Points

**Question:**

Is young-onset dementia associated with the risk of hospitalization for a motor vehicle crash injury in Taiwan?

**Findings:**

In this nationwide cohort study of 78 688 adults in Taiwan, individuals with young-onset dementia had a significantly higher risk of motor vehicle crash injury–related hospitalization than did those without dementia.

**Meaning:**

These findings suggest that transportation safety strategies should be planned for patients with young-onset dementia.

## Teaser

This cohort study assesses the risk of hospitalization for motor vehicle crash injuries among Taiwanese adults with young-onset dementia compared with age-matched individuals without dementia.

## Introduction

Dementia, a common cause of cognitive impairment, affects nearly 50 million people or 1% of the world’s population.^1^ Studies have demonstrated that the deficits associated with dementia influence the ability to drive and safely navigate traffic.^2,3,4,5^ Nevertheless, many patients with dementia continue to drive cars or ride bicycles or motorcycles to maintain their independence and active participation in the community.^6,7^ Thus, with the increasing number of dementia diagnoses worldwide,^1^ understanding and maintaining transport safety for patients with dementia constitute a major public health issue.

Several studies have explored the association between dementia and the risk of motor vehicle crash injury (MVCI), with some studies demonstrating that patients with dementia have a 2 to 18 times higher risk of MVCI compared with the general population.^8,9,10,11^ The results of these studies provide a better understanding of risks of MVCI and related outcomes. However, numerous studies on this topic have used self-reported traffic crashes,^8,9,10,11^ regional data with relatively small sample sizes,^8,9,10,11,12,13,14,15,16^ short follow-up periods,^8,10,11,13,15,16,17^ no distinction of the crash party (driver, pedestrian, or passenger)^17,18,19^ or vehicle class (such as cars, motorbikes, or bicycles),^8,9,10,11,12,13,14,15,16,17,18,19,20,21,22,23,24,25^ and no consideration of the length of time since dementia diagnosis.^10,11,13,15,16,17,18,19,22,24,25^ This has resulted in inconclusive and probably biased risk estimates. In addition, most of these studies have focused on elderly adults^8,9,10,11,12,13,14,15,17,18,21,22,23^; studies of the risk of MVCI in younger patients with dementia are limited.^16,18,19,24,25^

Considering the aforementioned method and data limitations with regard to this issue, further research is needed to explore the association between young-onset dementia and risk of MVCI. Accordingly, we conducted a nationwide, population-based cohort study in Taiwan to investigate the association between dementia and hospitalization for MVCI in adults aged 40 to 64 years. Furthermore, we evaluated this association in subgroups of different vehicle types, crash parties, injury types, and injury severity levels and for different periods of follow-up after dementia diagnosis.

## Methods

In this nationwide, population-based, retrospective cohort study, data from January 1, 2003, to December 31, 2015, were retrieved from Taiwan’s Police-Reported Traffic Accident Registry (PTAR) and National Health Insurance Research Database (NHIRD). The institutional review board of Jen-Ai Hospital approved the study protocol and its ethical aspects. All data in the NHIRD and PTAR were anonymous and retrospectively retrieved; thus, the need for informed consent was waived. This study followed the Strengthening the Reporting of Observational Studies in Epidemiology (STROBE) reporting guideline. Data were analyzed between March 25 and October 22, 2021.

### Data Source

Vehicle crashes in the PTAR were investigated by local police departments in accordance with Taiwan’s regulations governing road traffic accidents. Information on vehicle crashes between January 1, 2003, and December 31, 2015, was obtained from the PTAR. A completed PTAR crash report form includes profiles for both the accident and the victims.^26^ The crash profile includes details on the date and cause of a crash, and the victim profile records information about the vehicles and the victims, including vehicle types and whether the victims were drivers, riders, passengers, or pedestrians. Details of the PTAR have been described in a previous study.^27^

To obtain more reliable data on injury severity, the PTAR data were crossmatched with clinical data from the NHIRD using each individual’s personal identification number. The NHIRD was established in 1995 when the universal National Health Insurance program was launched in Taiwan. Since 1999, more than 99% of the Taiwanese population has been enrolled in the National Health Insurance program.^28^ To confirm the accuracy of the claims data, the National Health Insurance Administration performs quarterly expert reviews on the claims from every hospital and clinic.^28^ Therefore, the information on the NHIRD is regarded as complete and accurate.

### Study Design and Population

We included participants aged 40 to 64 years with at least 3 outpatient claim records containing *International Classification of Diseases, Ninth Revision, Clinical Modification (ICD-9-CM)* codes for an irreversible dementia-related diagnosis (290, 291, 294, 331, and 046.1) between 2006 and 2012. The criterion of at least 3 claims was adopted to increase the validity of dementia identification. In addition, the first and last outpatient visits with records containing these codes were required to be at least 90 days apart to avoid the accidental inclusion of miscoded patients. These criteria for identification of dementia were used in previous studies on dementia using Taiwan’s NHIRD.^29,30^ The *ICD-9-CM* rather than the *International Statistical Classification of Diseases and Related Health Problems, Tenth Revision* was used to identify dementia cases because it was used to code and classify disease data in the NHIRD until 2016.^31^ For the group with dementia, we included individuals receiving a first-time diagnosis of dementia between 2006 and 2012 and excluded those with a dementia diagnosis before 2006. The date of initial diagnosis was set as the index date. We further excluded patients who had been hospitalized for an MVCI before the index date and those who were younger than 40 years before the index date. Patients younger than 40 years were excluded because young-onset dementia more commonly occurs from 40 to 64 years of age.^32^

For the group without dementia (reference group), we randomly selected individuals with no dementia diagnosis throughout the study period; the exclusion criteria for this cohort were the same as those used for the group with dementia. The 2 groups were frequency matched in a 1:1 ratio by age (within a 5-year span), sex, and index year, as implemented in other studies,^33,34^ because these characteristics have been recognized as confounders for associations between traffic injuries and dementia.^18,21,35^ For the group without dementia, the index date was January 1 of each year between 2006 and 2012.

### Baseline Characteristics

Baseline characteristics identified at the index date included sex, age, salary-based insurance premium, urbanization level of the individual’s township, and comorbidities that have been previously shown to be associated with traffic crashes.^36,37,38,39,40,41^ Age was also considered a stratification variable, with each stratum comprising a 5-year interval. The salary-based insurance premium was regarded as an indicator of an individual’s socioeconomic status, considering that a beneficiary’s personal income is a determinant for the amount of insurance premium in the National Health Insurance program in Taiwan.^42^ The urbanization level of each township in Taiwan was developed and defined by Liu et al,^43^ who used a cluster analysis based on squared Euclidean distance and a minimum variance method. Indicators used for calculating scores in the cluster analysis included population density, proportion of residents with a college education or higher, proportion of people older than 65 years, proportion of the agricultural workforce, and number of physicians per 100 000 population. Accordingly, townships were divided into 7 clusters originally, with 1 representing the most urbanized and 7 the least. In our study, the clusters were recategorized from 7 to 3 levels: urban (levels 1-2), suburban (3-4), and rural (5-7).^43^ Baseline comorbidities, observed within 1 year before the index date, included Parkinson disease, stroke, diabetes, anxiety disorders, depressive disorders, bipolar disorders, alcohol-related disorders, other substance-related disorders, insomnia, and cataracts.

### Outcome and Stratification Variables

The outcome measure was the occurrence of MVCI-related hospitalization (*ICD-9-CM* codes E810-E829), with 1 as the event occurring and 0 as not occurring. The number of events in the whole sample or a subgroup was then a count variable. Stratification variables including the type of injury, mode of transport, and injury severity were further considered to assess various risks of MVCI-related hospitalization among subgroups. The types of injury were determined by the *ICD-9-CM* codes in the NHIRD (eTable in the Supplement), and the modes of transport were defined by reports in the PTAR. The Injury Severity Score (ISS) was used to evaluate the severity of the injury among inpatients. The ISS ranges from 0 to 75, with an ISS of 16 or higher indicating major trauma.^44,45^ Calculation of the ISS relied on the severity of injury to various body regions according to *ICD-9-CM* codes and was implemented using the R package (R Project for Statistical Computing) that follows the scoring method proposed by ICD Programs for Injury Categorization.^46^

### Statistical Analysis

Distributions of baseline characteristics between the groups with and without dementia were first assessed. Study participants were followed up from the index date to the date of hospitalization for an MVCI, withdrawal from the National Health Insurance, or the end of year 2015, whichever came first. The incidence densities of overall, age-specific, and cause-specific MVCI-related hospitalization were calculated as the number of events (MVCI-related hospitalizations) by the total observed person-years. The 95% CIs were then calculated assuming the events followed Poisson distribution.^47^ To assess the association between dementia and MVCI-related hospitalization, multivariable Cox proportional hazards regression models were constructed to estimate adjusted hazard ratios (HRs) and their 95% CIs. The proportional hazards assumption was visually inspected based on log-minus-log plots, and no considerable violation was observed. Furthermore, in addition to the overall estimate (10 years of follow-up), we performed separate analyses for 1, 3, 5, and 7 years of follow-up. A 2-sided *P* < .05 was considered statistically significant. All statistical analyses were performed using SAS, version 9.4 (SAS Institute Inc). The analyses were performed at the Health and Welfare Data Science Centre supervised by the Ministry of Health and Welfare, Taiwan.

## Results

Of the 78 688 participants included in the study, with 39 344 in each cohort, 47 034 (59.8%) were male; the mean (SD) age was 54.5 (7.4) years. Compared with the group without dementia, the group with dementia had a lower median insurance premium and a lower proportion of participants living in an urban area but a higher prevalence of medical comorbidities (Table 1). The incidence density of MVCI-related hospitalization was 45.58 per 10 000 person-years (95% CI, 42.77-48.39 per 10 000 person-years) in the group with dementia and 24.10 per 10 000 person-years (95% CI, 22.22-25.99 per 10 000 person-years) in the group without dementia, with an adjusted HR (aHR) of 1.83 (95% CI, 1.63-2.06) (Table 2). The Figure presents the model-based estimated survival curves, which revealed significantly different rates of MVCI-related hospitalization between the 2 groups. In addition, compared with the group without dementia, the group with dementia had a significantly higher risk of MVCI-related hospitalization in all age strata (Table 2). Table 3 shows that dementia was associated with an increased risk of hospitalization for an MVCI diagnosis of fracture, intracranial or internal injury, open wound, superficial injury or contusion, or other injuries. The highest aHR was observed in those with a diagnosis of intracranial or internal injury (2.44; 95% CI, 2.02-2.94). Participants in the group with dementia were at higher risk of MVCI-related hospitalization when they were motorcyclists, passengers, cyclists, or pedestrians; the highest aHR was for pedestrians (2.89; 95% CI, 2.04-4.11). Regardless of a higher or lower ISS, the group with dementia showed a significantly higher risk of MVCI-related hospitalization, with aHRs of 1.68 (95% CI, 1.48-1.90) for those with an ISS less than 16 and 2.90 (95% CI, 2.16-3.89) for those with an ISS of 16 or higher. In addition, the aHR for the MVCI-related hospitalization for patients with dementia was highest within the first year after diagnosis (aHR, 3.53; 95% CI, 2.50-4.98). The aHR was 2.13 (95% CI, 1.79-2.54) within 3 years after diagnosis, 1.91 (95% CI, 1.67-2.19) within 5 years, and 1.87 (95% CI, 1.65-2.11) within 7 years (Table 4).

**Table 1.** Baseline Characteristics of Study Participants Recruited Between 2006 and 2012

Variable	Participants, No. (%) (N = 78 688)^a^	
	Dementia (n = 39 344)	No dementia (n = 39 344)
Sex		
Female	15 827 (40.2)	15 827 (40.2)
Male	23 517 (59.8)	23 517 (59.8)
Age, y		
40-44	5315 (13.5)	5315 (13.5)
45-49	5782 (14.7)	5782 (14.7)
50-54	6463 (16.4)	6463 (16.4)
55-59	8261 (21.0)	8261 (21.0)
60-64	13 523 (34.4)	13 523 (34.4)
Salary-based insurance premium, NTD^b^		
First tertile	15 990 (40.6)	11 113 (28.2)
First to <second tertile	12 769 (32.5)	12 121 (30.8)
≥Second tertile	10 583 (26.9)	16 110 (41.0)
Township urbanization		
Rural	5144 (13.7)	3663 (9.7)
Suburban	12 064 (32.0)	11 284 (29.7)
Urban	20 543 (54.3)	22 980 (60.6)
Comorbidities		
Parkinson disease	1326 (3.4)	51 (0.1)
Stroke	10 298 (26.1)	857 (2.18)
Diabetes	7779 (19.8)	3810 (9.7)
Anxiety disorders	7825 (19.9)	1723 (4.4)
Depressive disorders	5894 (15.0)	640 (1.6)
Bipolar disorders	1132 (2.9)	93 (0.2)
Alcohol-related disorders	3076 (7.8)	42 (0.1)
Other substance-related disorders	899 (2.3)	79 (0.2)
Insomnia	7650 (19.4)	2141 (5.4)
Cataracts	1731 (4.4)	687 (1.8)

Abbreviation: NTD, New Taiwan Dollars.

^a^ Any inconsistencies between the total sample size and the total number of participants at all levels of a variable are owed to missing information. Except for sex and age (matching variables), distributions of all other variables were significantly different (*P* < .001) between the 2 groups.

^b^ First tertile, 19 200; second tertile, 26 700.

**Table 2.** Overall and Age-Specific Incidence Densities of Hospitalization for Motor Vehicle Crash Injury

Variable	Group with dementia			Group without dementia			aHR (95% CI)^b^	*P* value
	Events, No. (%)	Person-years	ID, per 10 000 person-years (95% CI)^a^	Events, No. (%)	Person-years	ID, per 10 000 person-years (95% CI)^a^		
Overall	1013 (100.0)	222 239.8	45.58 (42.77-48.39)	628 (100.0)	26 0550.1	24.10 (22.22-25.99)	1.83 (1.63-2.06)	<.001
Age, y								
40-44	192 (19.0)	31 278.7	61.38 (52.70-70.07)	51 (8.1)	37 203.8	13.71 (9.95-17.47)	3.54 (2.48-5.07)	<.001
45-49	181 (17.9)	33 634.1	53.81 (45.97-61.65)	81 (12.9)	39 719.2	20.39 (15.95-24.83)	2.44 (1.80-3.30)	<.001
50-54	160 (15.8)	36 891.4	43.37 (36.65-50.09)	86 (13.7)	43 165.7	19.92 (15.71-24.13)	2.07 (1.53-2.79)	<.001
55-59	181 (17.9)	46 254.6	39.13 (33.43-44.83)	143 (22.8)	54 114.7	26.43 (22.09-30.76)	1.51 (1.17-1.96)	.001
60-64	299 (29.5)	74 180.9	40.31 (35.74-44.88)	267 (42.5)	86 346.6	30.92 (27.21-34.63)	1.37 (1.13-1.65)	.001

Abbreviations: aHR, adjusted hazard ratio; ID, incidence density.

^a^ Based on Poisson assumptions.

^b^ Based on a Cox proportional hazards regression model that adjusted for sex, age, salary-based insurance premium, urbanization, and comorbidities when estimating the overall ID; age was excluded from the model when estimating age-specific IDs.

**Figure.**
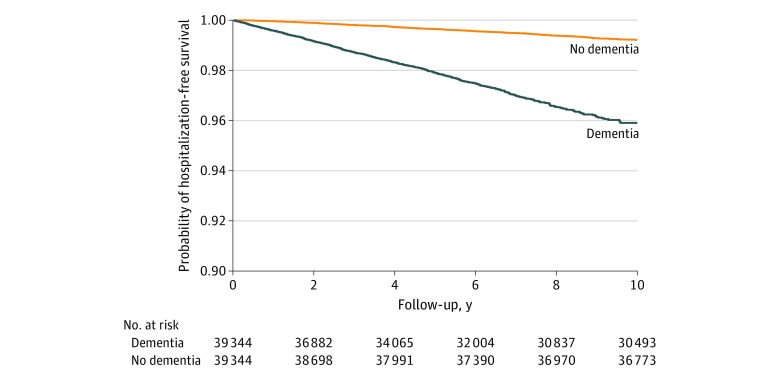
Probability of Hospitalization-Free Survival After Motor Vehicle Crash Injuries Among Individuals With and Without Dementia

**Table 3.** Associations Between Dementia and Risk of Hospitalization for MVCI According to Injury Type, Injury Severity Score, and Transport Mode

Variable	Group with dementia			Group without dementia			aHR (95% CI)^b^	*P* value
	Events, No. (%)	Person-years	ID, per 10 000 person-years (95% CI)^a^	Events, No. (%)	Person-years	ID, per 10 000 person-years (95% CI)^a^		
Total	1013 (100.0)	222 239.8	45.58 (42.77-48.39)	628 (100.0)	260 550.1	24.10 (22.22-25.99)	1.83 (1.63-2.06)	<.001
Type of injury								
Fracture	666 (65.7)	223 279.4	29.83 (27.56-32.09)	465 (74.0)	261 069.1	17.81 (16.19-19.43)	1.70 (1.48-1.95)	<.001
Dislocation	25 (2.5)	225 244.9	1.11 (0.67-1.54)	22 (3.5)	262 374.6	0.84 (0.49-1.19)	1.42 (0.72-2.78)	.31
Sprains and strains	21 (2.1)	225 238.2	0.93 (0.53-1.33)	12 (1.9)	262 396.5	0.46 (0.20-0.72)	1.72 (0.76-3.88)	.19
Intracranial or internal injury	448 (44.2)	224 049.1	20.00 (18.14-21.85)	210 (33.4)	261 807.3	8.02 (6.94-9.11)	2.44 (2.02-2.94)	<.001
Open wound	230 (22.7)	224 566.8	10.24 (8.92-11.57)	131 (20.9)	262 012.0	5.00 (4.14-5.86)	2.01 (1.57-2.58)	<.001
Superficial injury or contusion	354 (34.9)	224 184.8	15.79 (14.15-17.44)	231 (36.8)	261 676.5	8.83 (7.69-9.97)	1.77 (1.46-2.14)	<.001
Other and unspecified injuries of external causes	147 (14.5)	224 856.2	6.54 (5.48-7.59)	97 (15.4)	262 138.1	3.70 (2.96-4.44)	1.81 (1.35-2.43)	<.001
Transport mode								
Driver of motor vehicle	48 (4.7)	225 175.9	2.13 (1.53-2.73)	41 (6.5)	262 298.5	1.56 (1.08-2.04)	1.15 (0.72-1.84)	.56
Motorcyclist	683 (67.4)	223 288.0	30.59 (28.29-32.88)	461 (73.4)	261 065.6	17.66 (16.05-19.27)	1.67 (1.45-1.91)	<.001
Passenger	57 (5.6)	225 127.3	2.53 (1.87-3.19)	23 (3.7)	262 355.9	0.88 (0.52-1.23)	2.60 (1.53-4.42)	<.001
Pedal cyclist	79 (7.8)	225 096.6	3.51 (2.74-4.28)	45 (7.2)	262 297.7	1.72 (1.21-2.22)	2.35 (1.55-3.56)	<.001
Pedestrian	138 (13.6)	224 897.7	6.14 (5.11-7.16)	55 (8.8)	262 262.9	2.10 (1.54-2.65)	2.89 (2.04-4.11)	<.001
Other	8 (0.8)	225 311.3	0.36 (0.11-0.60)	3 (0.5)	262 427.4	0.11 (0.01-0.24)	0.87 (0.18-4.14)	.86
MVCI injury severity score^c^								
<16	816 (80.6)	222 739.0	36.63 (34.12-39.15)	549 (87.4)	260 762.9	21.05 (19.29-22.81)	1.68 (1.48-1.90)	<.001
≥16	197 (19.4)	224 832.2	8.76 (7.54-9.99)	79 (12.6)	262 219.8	3.01 (2.35-3.68)	2.90 (2.16-3.89)	<.001

Abbreviations: aHR, adjusted hazard ratio; ID, incidence density; MVCI, motor vehicle crash injury.

^a^ Based on Poisson assumptions.

^b^ Based on Cox proportional hazards regression adjusted for sex, age, salary-based insurance premium, urbanization, and comorbidities.

^c^ Range, 0-75, with a score of 16 or higher indicating major trauma.

**Table 4.** Association Between Dementia and Risk of Hospitalization for Motor Vehicle Crash Injury, by Follow-Up Period

Follow-up period, y	Group with dementia			Group without dementia			aHR (95% CI)^b^	*P* value
	Events, No. (%)	Person-years	ID, per 10 000 person-years (95% CI)^a^	Events, No. (%)	Person-years	ID, per 10 000 person-years (95% CI)^a^		
≤1	183 (18.1)	38 976.7	46.95 (40.15-53.75)	51 (8.1)	39 191.3	13.01 (9.44-16.58)	3.53 (2.50-4.98)	<.001
3	523 (51.5)	112 659.6	46.42 (42.44-50.40)	237 (37.7)	116 473.8	20.35 (17.76-22.94)	2.13 (1.79-2.54)	<.001
5	773 (76.3)	170 173.6	45.42 (42.22-48.63)	410 (65.3)	183 955.7	22.29 (20.13-24.45)	1.91 (1.67-2.19)	<.001
7	937 (92.5)	204 808.5	45.75 (42.82-48.68)	532 (84.7)	230 574.2	23.07 (21.11-25.03)	1.87 (1.65-2.11)	<.001
Overall (≤10)	1013 (100)	222 239.8	45.58 (42.77-48.39)	628 (100)	260 550.1	24.10 (22.22-25.99)	1.83 (1.63-2.06)	<.001

Abbreviations: aHR, adjusted hazard ratio; ID, incidence density.

^a^ Based on Poisson assumptions.

^b^ Based on Cox proportional hazards regression adjusted for sex, age, salary-based insurance premium, urbanization, and comorbidities.

## Discussion

To our knowledge, this is the first nationwide, longitudinal, registry-based cohort study to examine the risk of MVCI-related hospitalization in patients with young-onset dementia. During the 10-year follow-up, participants with dementia had a significantly higher risk of MVCI-related hospitalization compared with those without dementia, particularly within a shorter period after dementia was diagnosed. In addition, the risk of MVCI-related hospitalization associated with dementia differed according to injury type, transport mode, and injury severity.

Our findings are consistent with those of other studies suggesting a higher risk of MVCI in individuals with dementia than in the general population.^8,9,10,11^ Several mechanisms of dementia may increase such a risk. First, dementia can lead to cognitive impairment in memory, attention, executive function, insight and judgment, problem-solving skills, hand-eye coordination, reaction time, and visuospatial abilities.^48^ These functions are necessary for safe walking and driving.^48^ Second, certain medical comorbidities such as diabetes and mental disorders are more prevalent in individuals with dementia,^49^ which may limit driving ability and be associated with an increased crash risk.^36,37^ Third, patients with dementia tend to have lower socioeconomic status,^50^ which may be associated with an increased number of traffic violations^51^ or dangerous driving behaviors (such as running red lights or driving under the influence of alcohol).^52^ Fourth, patients with dementia are more likely to live in less-urban areas than those who do not have dementia.^53^ They might have to travel greater distances, which is associated with greater risk of traffic accidents.^54^ However, our study demonstrated that there was an independent association between dementia and the risk of MVCI-related hospitalization after model adjusting. Therefore, other dementia-related factors may exist and be associated with MVCI. For example, patients with dementia tend to live in lower socioeconomic areas with poor road infrastructure and road surface conditions,^55^ which are associated with an increased risk of MVCI.^54^ Therefore, the relatively high risk of MVCI for patients with dementia could be confounded by their increased exposure to poor road conditions in lower socioeconomic areas.

Our results also showed that pedestrians with dementia had the highest risk of MVCI-related hospitalization. This finding is consistent with a previous study that found that moderate to high numbers of neurofibrillary tangles, which are among the neuropathologic hallmarks of Alzheimer disease, were more commonly associated with pedestrian crashes than with other causes of injury.^56,57^ Of note, our study also demonstrated that patients with dementia had a higher risk of MVCI-related hospitalization when they were passengers of a vehicle. This might be because patients with dementia have difficulty controlling their emotions and easily become disoriented,^3,58^ which may distract the driver and increase the risk of MVCI. In addition, for motorcyclists or pedal cyclists, dementia was also significantly associated with MVCI-related hospitalization. Patients with dementia tend to have a lower income level^50^ and therefore lower car ownership rates, which may be a factor in the association between dementia and the risk of MVCI.

Patients with dementia had a higher risk of an MVCI diagnosis of intracranial or internal injury than did those without dementia. Reasons for this finding remain unclear, but it might be associated with the mode of transport. A previous study found that head injury is the most common form of MVCI in pedestrians, accounting for most severe injuries.^59^ Consistent with this finding, our observation that pedestrians with dementia had the highest risk of MVCI-related hospitalization might explain why patients with dementia also had the highest risk of receiving an MVCI-related diagnosis of intracranial or internal injury. In addition, our study showed that the risk of MVCI-related hospitalization for severe injuries was highest among patients with dementia. This may be related to symptoms of dementia, such as impaired cognitive function,^48^ or to an increased number of comorbidities.^49^

Our study revealed that patients with dementia in different age strata and follow-up periods had an increased risk of MVCI-related hospitalization. The comparatively higher risks were found particularly in younger participants with dementia and within a shorter period of follow-up after diagnosis of dementia. These results are consistent with those of a previous study showing that the number of traffic crashes among participants with dementia was highest within the first 3 years of dementia onset and that the incidence density of traffic crashes decreased with age in participants with dementia.^9^ Empirical data have shown that cognitive function,^60,61^ older age,^61^ and disease duration and severity^9,61,62^ are associated with driving cessation among patients with dementia. Research suggested that approximately 50% of drivers with dementia stopped driving completely within 3 years after dementia was diagnosed, perhaps partly because of the increasing severity of the disease.^9^ Another study revealed that patients with dementia who were younger tended to have a lower proportion of driving cessation,^61^ which may explain why younger patients with dementia have less cognitive function decline and thus less driving impairment.^6,63^ Consequently, younger patients with dementia may have greater exposure to traffic environments, and this higher level of exposure to traffic at a younger age and earlier stage of the disease may explain the results of our study.

### Strengths and Limitations

This study has strengths. First, we obtained a large sample from 2 national data sets, which provided reliable data and enabled us to conduct highly representative and specific analyses. The PTAR provides comprehensive data on traffic crashes (including vehicle types). Therefore, our results may be more reliable than those of studies using only *ICD-9-CM* E codes to identify the causes of injury and the vehicle used. Second, we conducted a retrospective cohort study for 10 years of follow-up, which is longer than that of several other studies.^8,10,11,13,15,16,17^ Third, we estimated the risk of MVCI-related hospitalization for people initially diagnosed as having dementia rather than for those with prevalent dementia to minimize variations associated with disease severity. Fourth, we measured the risk of MVCI-related hospitalization for different strata, including types of injury, mode of transport, and injury severity, to provide further information.

The study also has limitations. First, we relied on the disease codes in the claims data to select participants with dementia, which might have led to potential misclassification. However, the criteria of at least 3 outpatient claim records and more than 90 days between the first and last dementia-related visits should mitigate disease misclassification. Second, some diagnoses for dementia may be delayed or underestimated, which might have limited our risk estimates. To address this issue, we conducted a sensitivity analysis using the 1-year period before dementia onset as a baseline, which produced similar results. Third, the PTAR records only injuries from crashes reported to the police at the scene for injury compensation claims. Therefore, injuries observed days after crashes were not included in our data, which might have led to an underestimation of the incidence of MVCIs. However, this was unlikely to affect our results because MVCI-related hospitalization was the research outcome, which allowed us to include only people with sufficient injuries to warrant crash compensation. Fourth, because some information from the PTAR and NHIRD was unavailable, some factors associated with traffic injuries, such as driving experience, traffic volume, or lifestyles (eg, sleeping habits, coffee consumption), could not be considered, which may have led to residual confounding bias.

## Conclusions

In this cohort study, during 10 years of follow-up, participants with dementia had an approximately 2-fold greater risk of MVCI-related hospitalization compared with participants without dementia. Moreover, the risk of MVCI-related hospitalization associated with dementia was highest within the first year after diagnosis of dementia and among participants who were pedestrians at the time of crashes. These findings suggest a need for the planning of strategies to prevent transportation crashes among patients with young-onset dementia.

## Notes

Supplement. **eTable.** *ICD-9-CM* List of the Types of Injury Resulting in MVCI-Related Hospitalization
