# Supplementary material for: Association Between Young-Onset Dementia and Risk of Hospitalization for Motor Vehicle Crash Injury in Taiwan
Source: JAMA Netw Open. 2022 May 5;5(5):e2210474. doi: 10.1001/jamanetworkopen.2022.10474 (PMC9073564; doi:10.1001/jamanetworkopen.2022.10474)
Supplement: Supplement. — eTable. ICD-9-CM List of the Types of Injury Resulting in MVCI-Related Hospitalization [file jamanetwopen-e2210474-s001.pdf]

Supplementary Online Content

Liu CC, Liu CH, Chang KC, Ko MC, Lee PC, Wang JY. Association between young-onset dementia and risk of hospitalization for motor vehicle crash injury in Taiwan. *JAMA Netw Open*. 2022;5(5):e2210474. doi:10.1001/jamanetworkopen.2022.10474

eTable. *ICD-9-CM* List of the Types of Injury Resulting in MVCI-Related Hospitalization

This supplementary material has been provided by the authors to give readers additional information about their work.

© 2022 Liu CC et al. *JAMA Network Open*.

eTable ICD-9-CM list of the types of injury resulting in MVCI-related hospitalization

Type of injury	ICD-9-CM
Fracture	800-829
Dislocation	830-839
Sprains and strains	840-848
Intracranial/ internal injury	850-869
Open wound	870-897
Superficial injury/ contusion	900-924
Other and unspecified effects of external causes	Crushing (925-929)、Foreign body entering through orifice (930-939)、Burn (940-949)、Injury to nerves and spinal cord (950-957)、Poisoning (960-989)、Other injuries (958-959, 990-999)

© 2022 Liu CC et al. *JAMA Network Open*.
